# RNA N^6^-methyladenosine modification is required for miR-98/MYCN axis-mediated inhibition of neuroblastoma progression

**DOI:** 10.1038/s41598-020-64682-1

**Published:** 2020-08-12

**Authors:** Junmei Cheng, Lingling Xu, Liqiang Deng, Lan Xue, Qingmei Meng, Furong Wei, Jinghua Wang

**Affiliations:** 1 Department of Pathology and Central Laboratory of Heze Medical College, Heze, 274000 Shandong China; 2 Department of Pediatrics, General Hospital of Ningxia Medical University, Ningxia, China; 3 Department of Pediatric Otolaryngology, the First People’s Hospital of Chenzhou, Chenzhou, 423000 China; 4 Department of Stomatology and Central Laboratory of Heze Medical College, Heze, 274000 Shandong China; 5 Department of Gastroenterology, Central Hospital of Haining, Zhejiang, China; 6 Department of Pediatric of Rheumatology, Immunology and Allergy, The First hospital of Jilin University, Changchun, 130021, Jilin, China

**Keywords:** Cell biology, Cell migration, Biomarkers

## Abstract

Neuroblastoma (NB) is one of the most common malignant tumors of the sympathetic nervous system in childhood. NB severely threatens patient’s health and life. However, more effective diagnosis and treatment methods are badly needed in clinics all over the world. MYCN is well recognized as a genetic biomarker of high risk and poor outcome in NB. miRNAs are small RNAs and miR-98 involved in the pathogenesis of various cancers. The role and mechanism of miR-98 in NB remains to be investigated. Here we found that miR-98 was decreased in human MYCN-high-expression NB tissues, and its down-regulation was associated with poor prognosis of NB. Over-expression of miR-98 inhibited cell proliferation, migration and invasion of NB cells. The analysis by employing the software of miRanda predicted the possible binding sites of miR-98 in the 3′-UTR of *MYCN*, and experimental data illustrated that miR-98 directly bound to MYCN 3′-UTR and decreased MYCN expression. Over-expression of MYCN rescued the decreased malignant phenotype caused by over-expression of miR-98 in NB. N^6^-methyladenosine modification in 3′-UTR of MYCN promoted its interaction with miR-98. The data collectively demonstrated that RNA m^6^A modification was required for miR-98/MYCN axis-mediated inhibition of neuroblastoma progression, and miR-98 might be novel targets for NB detection and treatment.

## Introduction

Neuroblastoma (NB) is one kind of embryonal extracranial solid tumor, and it is also one of the malignant tumors in the nervous system in children^1^. The clinical characteristic of NB is heterogeneity^2,3^. NB severely threatens patient’s health and life. The NB of high-stage is a high-risk pediatric malignancy. In addition, the median age of the NB patients is about 17 months at the time of diagnosis^4,5^. However, the pathogenesis of NB remains to be further investigated and more effective diagnosis and treatment methods are badly needed in clinics all over the world. Thus, novel effective targets for diagnosis and treatment of NB are badly needed in clinics.

*MYCN* gene belongs to the MYC family and it encodes a protein with a typical domain of basic helix-loop-helix (bHLH). MYCN is located in nucleus and it can bind DNA after dimerizing with another bHLH protein^6,7^. In addition, *MYCN* gene is an important MYC member of the oncogene family. When mutated, the oncogene of the *MYCN* gene can cause normal cells to be cancerous. The *MYCN* gene regulates cell growth, cell proliferation and cell apoptosis. Thus, the *MYCN* gene is closely related to a variety of cancers, especially NB^8,9^. Specifically, MYCN is a genetic biomarker of NB^10^. However, the mechanism of the *MYCN* gene in NB remains to be further studied.

miRNAs are small RNAs without coding potential but with about 20–22 nucleotides which can down-regulate gene expression post-transcriptionally by targeting to its target^11,12^. Increasing evidence has elucidated that miR-98 is involved in the pathogenesis of various cancers^13^. The objective of this research was to investigate whether miR-98 participates in MYCN’s function in NB. The underlying mechanism by which N^6^-methyladenosine (m^6^A) modification affected miR-98/MYCN interaction was also explored in the present investigation.

## Materials and methods

### Research subjects

In this study, 60 patients with NB and 60 paracancerous controls were collected according to the inclusion and exclusion criteria. The clinical data of the subjects are shown in Table 1. Approval for the experiment involving human subjects was obtained from the institutional review board of Jilin University. The informed consent of the subjects was provided according to the declaration of Helsinki as described previously^14^. The neuroblastoma tissues of the primary NB children were defined as reported previously^15^. The paracancerous tissues were the matched adjacent non-cancerous tissue samples, which were collected from a segment of the resected samples located at more than 5 cm away from the primary neuroblastoma tissue site after they died^15^. NB tissues and paracancerous control tissues were collected and subjected into western blot and real-time PCR analysis.

**Table 1:** Clinical data of the patients with NB and expression of miR-98.

Chinicopathological characteristics	Total	miR-98 high-expression group (n = 30)	miR-98 low-expression group (n = 30)	*P* value
**Gender**				
male	36	19	17	0.1417
female	24	11	13	
**Age**				
< = 40	38	18	20	0.2484
>40	22	12	10	
**Grade**				
INSSA	27	19	8	0.0663
INSSB	21	9	12	
INSSC	12	2	10	
**Lymph node metastasis**				
Positive	34	10	24	<0.001
Negative	26	20	6	
**OpticNerveInfiltration**				
yes	34	11	23	
no	23	19	7	<0.002

### Reagents, antibodies and cell lines

In this study, RNA extraction kits and CCK-8 kits were bought from Sigma. Primers targeting *MYCN* and miR-98 were designed and produced by Shanghai Gene Pharma Co.,Ltd. The anti-MYCN antibody was purchased from Sigma. The cell lines of SH-SY5Y, SK-N-AS, IMR-32 and SK-N-DZ were purchased from the American Type Culture Collection, Manassas, USA. All other reagents were from Merck.

### Real-time reverse transcription PCR (real-time PCR)

RNA was extracted from the tissues of NB and controls employing TRIzol. The RNA was reversely transcribed into cDNA by employing the kit (Qiagen, Hilden, Germany). The expression levels of *MYCN* and miR-98 were analyzed by using the one stop SYBR RT-PCR kit and examined by the 2^−ΔΔCt^ method. GAPDH served as the control.

### Western blotting

Cells or tissues of NB were lyzed in lysis buffer containing protease inhibitors (Thermo Fisher Scientific Inc). BCA kits were used for examination of protein concentrations. The cell lysates were then subjected into SDS-PAGE and western blotting analysis. After blocking with 5% milk for the transferred membrane containing proteins, primary antibodies against MYCN were used to incubate for 4 hours (h) at room temperature. After washing 3 times with PBST, the secondary antibody was employed to incubate with membrane for 2 h at room temperature. Finally, the ECL system was employed to show the relative expression of the targeted proteins^16^. GAPDH served as controls.

### Cell proliferation

In this study, cell proliferation was analyzed by using CCK-8 as described previously.

### Clone formation experiments

Cells with a concentration of 2 × 10^3^ cells/well were plated into 24-well plates. Cell medium was replaced every three days. The cells were washed with PBS 2 weeks after cell culturing, and then fixed with methanol for 5 min followed by incubating with 0.1% crystal violet for 6 min. Finally, the colonies were analyzed and counted manually.

### Transwell experiments

Cell invasion and migration were analyzed by using Transwell chambers (Corning Incorporated, Corning, NY) in the presence or absence of extracellular matrik (ECM). Cells with a concentration of 2 × 10^4^ cells/well were seeded into the upper chamber with fresh DMEM medium containing 10% FBS. After incubation for 24 h, the cells were fixed by employing 4% paraformaldehyde for 15 min and stained with 0.5% crystal violet for 6 min. Finally, the cells on the bottom of the membrane were counted and analyzed by a light microscope.

### Survival curve

**Figure 1:**
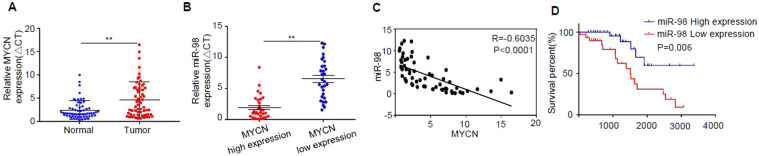
miR-98 is decreased in human MYCN-high-expression NB tissues, and its down-regulation is associated with poor prognosis of NB. (**A**) The expression levels of *MYCN* in 60 pairs of NB tissues and paracancerous control tissues were analyzed by q-RT-PCR. (**B**) Sixty cases of NB patients were divided into *MYCN* low-expression group (n = 30) and *MYCN* high-expression group (n = 30) according to the median value of the expression level of *MYCN*, and the expression of miR-98 in the *MYCN* low-expression group and the *MYCN* high-expression group was detected by real-time PCR. (**C**) The correlation between the expression of miR-98 and *MYCN* in the above 60 cases of NB was analyzed by Pearson correlation coefficient. (**D**) Evaluation of miR-98 low-expression group (n = 30) and miR-98 high-expression group (n = 30) was conducted by Kaplan-Meier survival curve, and the survival percent of the patients with NB was illustrated. Three independent experiments have been done. At least three samples were included in an independent experiment if it was not specified in the figures.

The survival curve was drawn by employing Graphpad software. Using the median value of miR-98 as the cutoff value, the overall survival time of the NB patients was shown in Fig. 1D.

### Luciferase reporter analysis

The predicted miR-98 binding sites on the 506–511 on 3′-UTR of *MYCN* was selected and sub-cloned into the psiCHECK-2 vector. The mutant-type and wild-type reporter of *MYCN* were produced by using the GeneArt Site-Directed Mutagenesis System. The cells were co-transfected with the constructed reporters. Dual-Luciferase Reporter System (Promega) was employed to analyze the luciferase activities 48 h after transfection.

### m^6^A-RIP

RNA immunoprecipitation assay was conducted by employing the RNA-binding protein immunoprecipitation kit (Millipore). Cell lysates were incubated with the RIP buffer containing magnetic beads conjugated with anti-MYCN antibody. IgG served as a control. The immunoprecipitated RNA was purified with Proteinase K. The purified RNA samples were subjected to real-time PCR analysis.

### Statistical analysis

All the data were analyzed by employing SPSS 16.0 software (SPSS Inc)and GraphPad PRIS version 5.01 (GraphPad Software, Inc). Data are shown as mean ± standard error of the mean. ANOVA and Student *t* test were used (*P < 0.05; **P < 0.01; ***P < 0.001). Three independent experiments have been done. At least three samples were included in an independent experiment if it was not specified in the figures.

## Results

### miR-98 was decreased in human MYCN-high-expression NB tissues, and its down-regulation was associated with poor prognosis of NB

MYCN is well recognized as a genetic biomarker of high risk and poor outcome in NB. miRNAs are small RNAs lacking protein-coding potential with a length of about 20–22 nucleotides, and miR-98 is involved in the pathogenesis of various cancers. However, the level of miR-98 in NB tissues and its relationship with *MYCN* remain to be investigated. The expression of miR-98 in NB tissues was firstly studied in this investigation. Sixty patients with NB and 60 paracancerous controls were collected according to the inclusion and exclusion criteria. The clinical data of the subjects are shown in Table 1.

Chi-square test was employed to analyze the correlation between miR-98 expression and clinical parameters in 60 patients with NB. miR-98 low-expression was closely related to lymph node metastasis, and high-grade INSS stage was closely related to optic nerve invasion, and the difference was statistically significant (*P* < 0.0001) (Table 1). However, miR-98 expression showed little relationship with age and sex.

Real-time PCR was employed to analyze the expression levels of *MYCN* and miR-98 and the data demonstrated that the expression level of *MYCN* in tumor tissues was significantly higher than that of normal control tissues (*P* < 0.001) (Fig. 1A).

Sixty cases of NB patients were divided into *MYCN* low-expression group (n = 30) and *MYCN* high-expression group (n = 30) according to the median value of the expression level of *MYCN*, and the expression of miR-98 in the *MYCN* low-expression group and the *MYCN* high-expression group was detected by real-time PCR. However, the level of miR-98 in the *MYCN*-high-expression group was significantly lower than that of the *MYCN* -low-expression group (Fig. 1B). The correlation between the expression of miR-98 and *MYCN* in the above 60 cases of NB was analyzed by Pearson correlation coefficient. Interestingly, the results revealed that there was a significant negative correlation trend between the expression of miR-98 and *MYCN* in the above 60 cases of NB, and the difference was statistically significant (*P* < 0.001, R = 0.6035) (Fig. 1C). In addition, the survival percent of the patients with NB in the *MYCN* low-expression group (n = 30) and the *MYCN* high-expression group (n = 30) was also studied and the data showed that the survival rate of the NB patients in the miR-98 low-expression group was significantly higher than that of the miR-98 high-expression group (*P* < 0.006) (Fig. 1D).

### Over-expression of miR-98 inhibited cell proliferation, cell migration and invasion of NB cells

**Figure 2:**
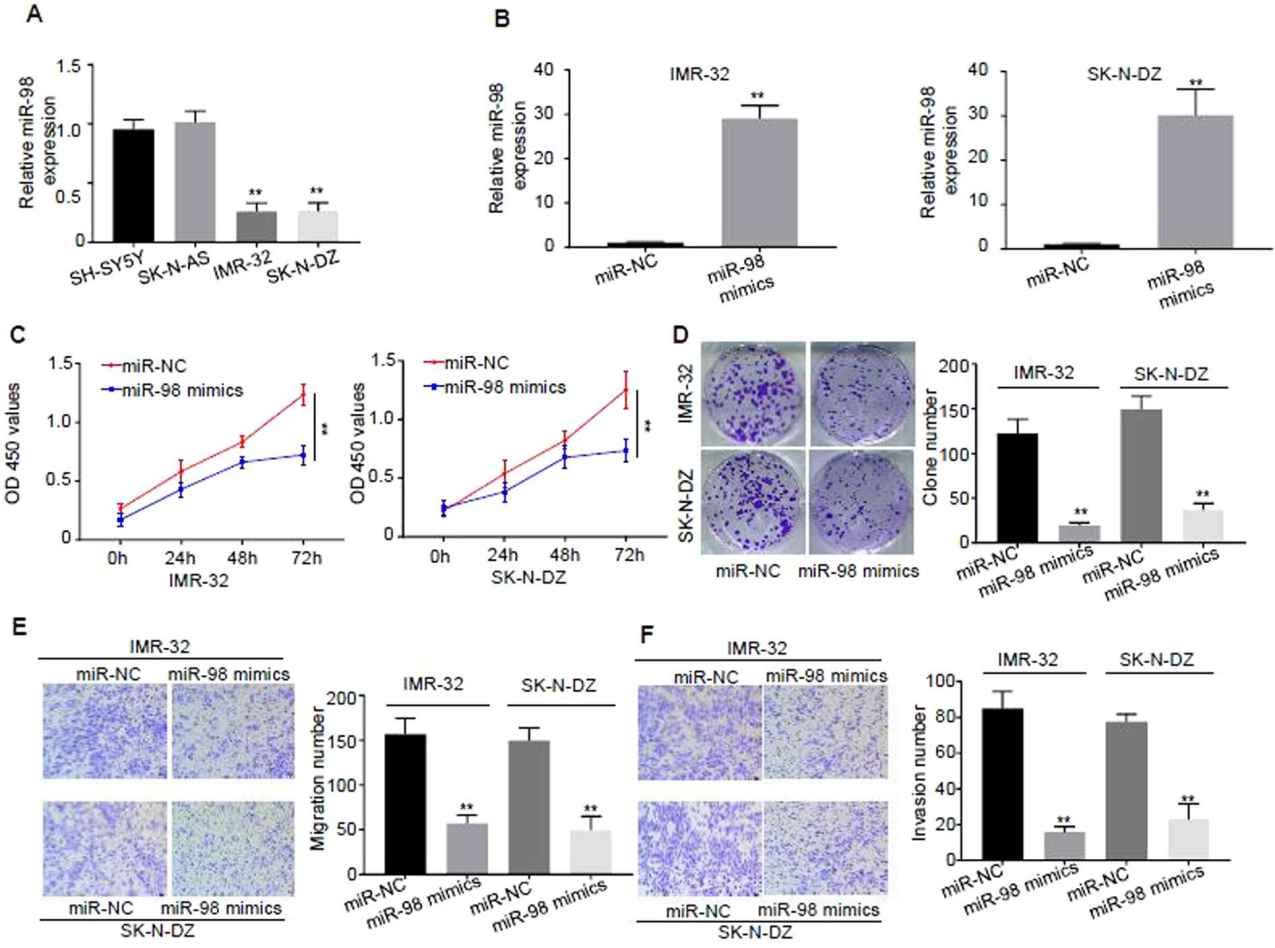
Over-expression of miR-98 inhibits proliferation, migration and invasion of NB cells. (**A**) Expression levels of miR-98 in NB cell lines of SH-SY5Y with normal copy of *MYCN*, SK-N-AS with normal copy of *MYCN*, IMR-32 with high copy of *MYCN*, and SK-N-DZ with high copy of *MYCN* were detected by real-time PCR. (**B**) The cells of IMR-32 and SK-N-DZ were transfected with miR-98 mimics and control miR-NC, and the transfection efficiency was analyzed by real-time PCR. (**C**) Cell proliferations of the cells of IMR-32 and SK-N-DZ transfected with miR-NC and miR-98 at time points of 0 h, 24 h, 48 h and 72 h were analyzed by CCK-8 at the light absorption of the wavelength of 450 nm. (**D**) Clone formation abilities of the cells of IMR-32 and SK-N-DZ transfected with miR-NC and miR-98 were analyzed by employing clone formation assay. (**E**) Migration abilities of the cells of IMR-32 and SK-N-DZ transfected with miR-NC and miR-98 were analyzed by using Transwell assay without EMC. (**F**) Invasive abilities of the cells of IMR-32 and SK-N-DZ transfected with miR-NC and miR-98 were analyzed by using Transwell assay with EMC. Three different experiments have been done. At least three samples were included in an independent experiment.

To further explore role of miR-98 in NB, several cell lines with different expression profiles of *MYCN* were selected. The data in Fig. 1C revealed that there was a significant negative correlation trend between the expression of miR-98 and *MYCN* in the patients with NB, which prompt us to test the correlation in cell lines. Figure 2A illustrated that the expression levels of miR-98 in SH-SY5Y with normal copy of *MYCN* and SK-N-AS cells with normal copy of *MYCN* were significantly higher than that of the IMR-32 cells with high copy of *MYCN* and SK-N-DZ cells with high copy of *MYCN* (*P* < 0.01).

Next, the cells of IMR-32 and SK-N-DZ were transfected with miR-98 mimics and control miR-NC to further verify the role of miR-98 in cell lines. The transfection efficiency was analyzed by real-time PCR and the results revealed that transfection of miR-98 mimics increased the expression of miR-98 by more than 25 times comparing with that of the controls, and the difference was statistically significant (*P* < 0.01) (Fig. 2B). Clone formation experiment was employed to detect cell proliferation by using CCK-8 kit. Figure 2C,D shows that cell proliferations of the cells of IMR-32 and SK-N-DZ transfected with miR-NC and miR-98 at time points of 0 h, 24 h, 48 h and 72 h inhibited clone formation of the cells (*P* < 0.01).

Migration abilities of the cells of IMR-32 and SK-N-DZ transfected with miR-NC and miR-98 were analyzed by using Transwell assay without extracellular matrik (EMC) and the results illustrated that over-expression of miR-98 significantly decreased the migration of the cells (*P* < 0.01) (Fig. 2E). Invasive abilities of the cells of IMR-32 and SK-N-DZ transfected with miR-NC and miR-98 were analyzed by using Transwell assay with EMC. The results illustrated that over-expression of miR-98 significantly reduced the invasion ability of the cells (*P* < 0.01) (Fig. 2F).

Collectively, these data indicate that Over-expression of miR-98 inhibited cell proliferation, cell migration and invasion of NB cells.

### miR-98 directly bound to MYCN 3′-UTR and decreased MYCN expression

miRNAs are small RNAs lacking protein-coding potential and they can down-regulate gene expression post-transcriptionally by combining with complementary sequences of target mRNAs. Therefore, whether miR-98 can directly regulate expression of *MYCN* was investigated.

**Figure 3:**
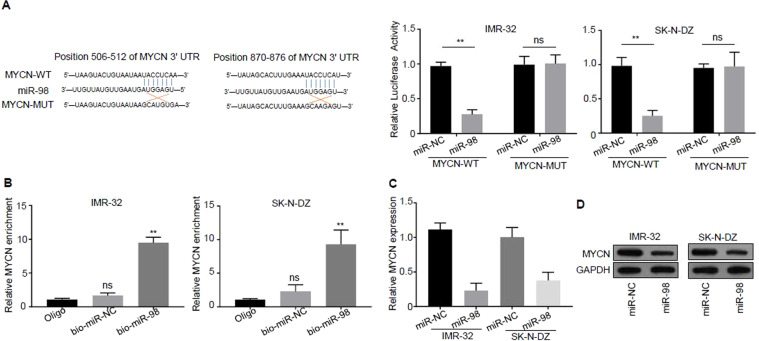
miR-98 directly binds to MYCN 3′-UTR and decreases MYCN expression. (**A**) The software of miRanda (http://www.microrna.org/) was employed to predict the possible binding sites of miR-98 in the 3′-UTR of *MYCN*: the regions of 506–511 and 870–875 in the 3′-UTR of *MYCN*, and the sequence is as follows: UACCUC. (**B**) Experimental confirmation of the interaction of miR-98 with *MYCN* by RNA pull-down. (**C**) The expression of *MYCN* mRNA in the cells of IMR-32 and SK-N-DZ transfected with miR-NC and miR-98 was analyzed by real-time PCR. (**D**) The expression of MYCN protein in the cells of IMR-32 and SK-N-DZ transfected with miR-NC and miR-98 was analyzed by western blotting. Three independent assays have been done. Three samples were included in an independent experiment if it was not specified in the figures.

The software of miRanda (http://www.microrna.org/) was employed to predict the possible binding sites of miR-98 in the 3′-UTR of *MYCN* and the results demonstrated that the regions of 506–511 and 870–875 in the 3′-UTR of *MYCN* were possible targets and the sequence was as follows: UACCUC (Fig. 3A). To further confirm the prediction results, these 2 sites of 506–511 and 870–875 in the 3′-UTR of *MYCN* were mutated and the luciferase reporter gene experiments were carried out in the cells of IMR-32 and SK-N-DZ. Figure 3A displayed that over-expression of miR-98 was able to inhibit luciferase activity in IMR-32 and SK-N-DZ cells compared to that of miR-NC, but the inhibitory effect disappeared only after mutation of the predicted miR-98 binding site 506–511 region, further suggesting that only the region of 506–511 in the 3′-UTR of *MYCN* was the binding target of miR-98.

The interaction of miR-98 with *MYCN* was verified by RNA pull-down assay. Specific primers containing the region of 506–511 on 3′-UTR of *MYCN* excluding the region of 870–875 were designed, and RNA pull-down experiments and real-time PCR were conducted in IMR-32 and SK-N-DZ cells. The data of real-time PCR showed that miR-98 probe could pull down more *MYCN* 3′- UTR compared with that of control oligo probe (*P* < 0.01) (Fig. 3B).

The mRNA expression of *MYCN* in the cells of IMR-32 and SK-N-DZ transfected with miR-NC and miR-98 was analyzed by real-time PCR. The results revealed that the expression level of the *MYCN* mRNA was inhibited by the over-expression of miR-98 compared with that of the control miR-NC, and the difference was statistically significant (*P* < 0.01) (Fig. 3C).

The expression of MYCN protein in the cells of IMR-32 and SK-N-DZ transfected with miR-NC and miR-98 was analyzed by western blotting. The results revealed that the expression level of the MYCN protein was reduced by the over-expression of miR-98 compared with that of the control miR-NC, and the difference was statistically significant (*P* < 0.01) (Fig. 3D).

Taken together, these results suggest that miR-98 directly bound to *MYCN* 3′-UTR and decreased MYCN expression

### Over-expression of MYCN rescued the decreased malignant phenotype caused by over-expression of miR-98 in NB

**Figure 4:**
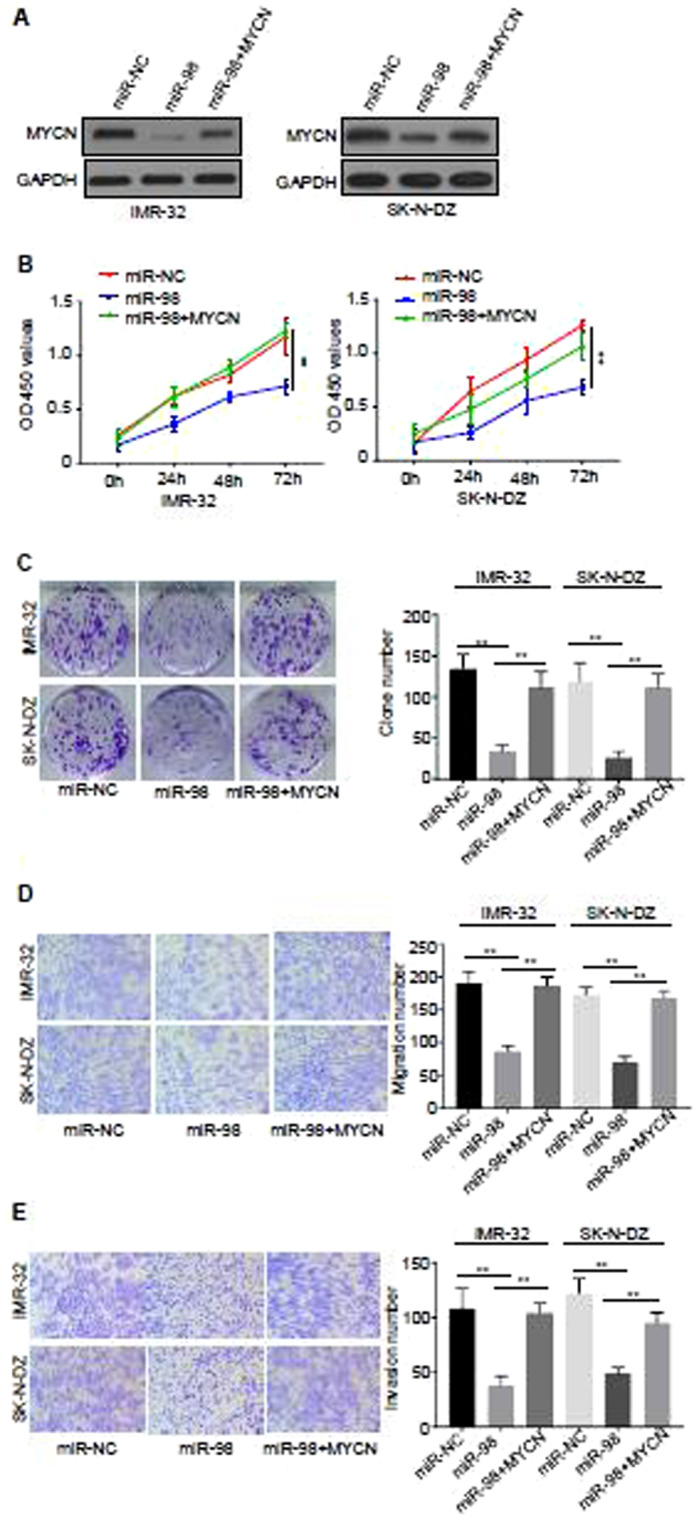
Over-expression of MYCN rescued the decreased malignant phenotype caused by over-expression of miR-98 in NB. (**A**) The expression of MYCN was detected by western blotting. Representative image from three distinct experiments was illustrated. (**B**) Cell proliferations of the cells of IMR-32 and SK-N-DZ transfected with miR-NC, miR-98 and miR-98+MYCN at time points of 0 h, 24 h, 48 h and 72 h were analyzed by CCK-8 at the light absorption of the wavelength of 450 nm. (**C**) Clone formation abilities of the cells of IMR-32 and SK-N-DZ transfected with miR-NC, miR-98 and miR-98+MYCN were analyzed by employing clone formation assay. (**D**) Migration abilities of the cells of IMR-32 and SK-N-DZ transfected with miR-NC, miR-98 and miR-98+MYCN were analyzed by using Transwell assay without EMC. (**E**) Invasive abilities of the cells of IMR-32 and SK-N-DZ transfected with miR-NC, miR-98 and miR-98+MYCN were analyzed by using Transwell assay with EMC. Three different experiments have been done. At least three samples were included in an independent experiment.

To further investigate the relationship between MYCN and miR-98, rescue experiment was carried out. Cell proliferations of the cells of IMR-32 and SK-N-DZ transfected with miR-NC, miR-98 and miR-98+MYCN at time points of 0 h, 24 h, 48 h and 72 h were analyzed and the data revealed that over-expression of miR-98 inhibited cell proliferation, and the proliferation ability was recovered significantly after co-transfection with *MYCN* in miR-98 over-expressed cells simultaneously (*P* < 0.01) (Fig. 4A).

Furthermore, clone formation abilities of the cells of IMR-32 and SK-N-DZ transfected with miR-NC, miR-98 and miR-98+MYCN were analyzed and the results demonstrated that over-expression of miR-98 significantly inhibited clone formation of the cells, and the clone formation ability increased partially after co-transfection with *MYCN* in miR-98 over-expressed cells simultaneously, and the difference was statistically significant (*P* < 0.01) (Fig. 4B).

In addition, migration abilities of the cells of IMR-32 and SK-N-DZ transfected with miR-NC, miR-98 and miR-98+MYCN were analyzed by using Transwell assay without EMC. The results illustrated that over-expression of miR-98 significantly decreased the migration of the cells, and the migration ability increased partially after co-transfection with *MYCN* in miR-98 over-expressed cells simultaneously, and the difference was statistically significant (*P* < 0.01) (Fig. 4C). Invasive abilities of the cells of IMR-32 and SK-N-DZ transfected with miR-NC, miR-98 and miR-98+MYCN were analyzed by using Transwell assay with EMC. The results showed that over-expression of miR-98 significantly reduced the invasion ability of the cells of of IMR-32 and SK-N-DZ, and the invasion ability increased partially after co-transfection with *MYCN* in miR-98 over-expressed cells simultaneously, and the difference was statistically significant (*P* < 0.01) (Fig. 4D).

These data collectively indicate that over-expression of MYCN rescued the decreased malignant phenotype caused by over-expression of miR-98 in NB.

### m^6^A modification in 3′-UTR of MYCN promotes its interaction with miR-98

Epigenetic modifications, especially m^6^A, are highly involved in gene expression and regulation. Therefore, whether m^6^A modification also participates in the gene expression and regulation of MYCN and miR-98 is explored in the present study.

**Figure 5:**
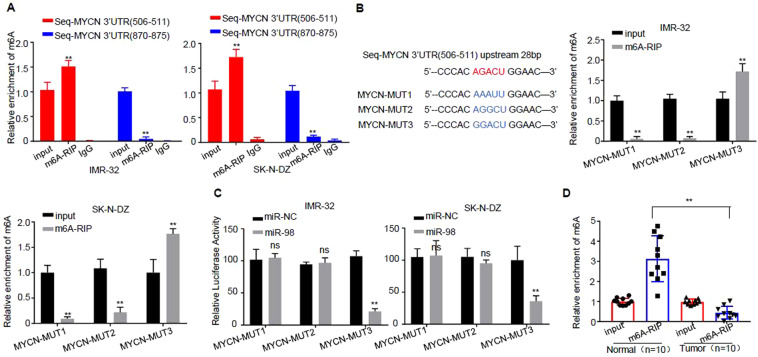
m^6^A modification in 3′-UTR of *MYCN* promotes its interaction with miR-98. (**A**) Experimental confirmation of the modification mechanism of the interaction of miR-98 with *MYCN* by m^6^A-RIP. IgG control sample was shown for control. (**B**) The 3′-UTR of *MYCN* sequence was analyzed. (**C**) The AGACU sequence was mutated into AAAUU, AGGCU and GGACU, and the luciferase reporter gene experiments were conducted in the cells of IMR-32 and SK-N-DZ, respectively. (**D**) m^6^A-RIP test was carried out in 10 pairs of NB tumor tissues and control tissues. real-time PCR was employed to detect the interaction of m^6^A with *MYCN*. Three independent experiments have been done. At least three samples were included in an independent experiment if it was not specified in the figures.

Two pairs of specific primers containing the regions of 506–511 and 870–875 on 3′-UTR of *MYCN* were designed, and m^6^A-RIP experiments and real-time PCR were conducted in IMR-32 and SK-N-DZ cells. The data of real-time PCR displayed that more than 10% m^6^A modification was enriched in the region of 506–511 on 3′-UTR of *MYCN*, while few m^6^A modification was enriched in the region of 870–875 on 3′-UTR of *MYCN*, compared with that of Input (Fig. 5A).

The 3′-UTR of *MYCN* sequence was further analyzed. It is found that there is an m^6^A modification motif in the upstream 28 bp position of the region of 506–511 (AGACU, m6A modification motif is RRACU; R stands for A or G)). The AGACU sequence was mutated into AAAUU, AGGCU and GGACU, and m^6^A-RIP experiments were conducted in the cells of IMR-32 and SK-N-DZ, respectively. real-time PCR detection was carried out in the pulled 3′-UTR of *MYCN* sequence. The results revealed that more than 10% m^6^A modification was enriched in the region of 506–511 on 3′-UTR of *MYCN* sequence (AGACU sequence was mutated to GGACU), whereas m^6^A modification disappeared when AGACU sequence was mutated to AAAUU or AGGCU (Fig. 5B).

The AGACU sequence was mutated into AAAUU, AGGCU and GGACU, and the luciferase reporter gene experiments were conducted in the cells of IMR-32 and SK-N-DZ, respectively. The data revealed that over-expression of miR-98 could inhibit the activity of luciferase in the cells of IMR-32 and SK-N-DZ (the sequence of AGACU was mutated to GGACU) compared with that of miR-NC, but when the sequence of AGACU was mutated to AAAUU and AGGCU, its inhibitory effect disappeared (Fig. 5C).

m^6^A-RIP test was carried out in 10 pairs of NB tumor tissues and control tissues. Real-time PCR was employed to detect the interaction of m^6^A with *MYCN*, and the results revealed that the region of 506–511 on 3′-UTR of *MYCN* was pulled down and m^6^A modification was more concentrated in the control tissues, and almost no m^6^A modification was found in NB tissues (Fig. 5D).

## Discussion

NB is one of the most common malignant tumors of the sympathetic nervous system in childhood, which severely threatens patient’s health and life. The high-stage NB is considered to be a high-risk pediatric malignancy with high death rate and poor prognosis. Therefore, more effective diagnosis and treatment methods are badly needed in clinics all over the world.

The main discoveries in the present investigation are as follows: (1) miR-98 was decreased in human *MYCN*-high-expression NB tissues, and its down-regulation was associated with poor prognosis of NB. (2) Over-expression of miR-98 inhibited cell proliferation, migration and invasion of NB cells. (3) The binding site of miR-98 was the region of 506–511 on the 3′-UTR of *MYCN*, and miR-98 directly bound to MYCN 3′-UTR and decreased MYCN expression. (4) Over-expression of *MYCN* rescued the decreased malignant phenotype caused by over-expression of miR-98 in NB. (5) N^6^-methyladenosine modification in 3′-UTR of MYCN promoted its interaction with miR-98. These data collectively demonstrated that RNA m^6^A modification was required for miR-98/MYCN axis-mediated inhibition of neuroblastoma progression, and miR-98 might be novel targets for NB detection and treatment.

MYCN is a well recognized genetic biomarker of high risk and poor outcome in NB and the data in this study further supports this point that the survival rate of the NB patients in the miR-98 low-expression group was significantly higher than that of the miR-98 high-expression group (*P* < 0.006) (Fig. 1D).

miRNAs are small RNAs and miR-98 is involved in the pathogenesis of various cancers, including NB. We have carried out a screening of miRNA assay in neuroblastoma progression and RNA N6-methyladenosine modification. The data demonstrated that miR-98 was highly involved in miR-98/MYCN axis-mediated inhibition of neuroblastoma progression by RNA N6-methyladenosine modification. For other miRNAs, there was no obvious phenotype in our system. Thus, miR-98 was selected in this investigation. The role and mechanism of miR-98 in NB remains were investigated in the current study. Interestingly, this study revealed that miR-98 was decreased in human *MYCN*-high-expression NB tissues, and its down-regulation was associated with poor prognosis of NB, indicating that miR-98 is involved in NB. On the other hand, over-expression of miR-98 inhibited cell proliferation, migration and invasion of NB cells, further supporting the role of miR-98 in NB. The analysis by employing the software of miRanda illustrated that the region of 506–511 on the 3′-UTR of *MYCN*, and miR-98 directly bound to MYCN 3′-UTR and decreased MYCN expression, whereas over-expression of MYCN rescued the decreased malignant phenotype caused by over-expression of miR-98 in NB, indicating that miR-98 indeed participates in NB by regulating cell proliferation, cell clone formation and migration. RIP analysis further revealed that N^6^-methyladenosine modification in 3′-UTR of *MYCN* promoted its interaction with miR-98, suggesting that RNA m^6^A modification was required for miR-98/MYCN axis-mediated inhibition of neuroblastoma progression, and miR-98 might be novel targets for NB detection and treatment.

There are several limitations in the present study: (1) The number of the research subjects are limited, so more patients should be included to further verify the conclusion. (2) Although miR-98/MYCN axis showed significant effects on cells proliferation and clone formation *in vitro*, whether it is the case *in vivo* remains to be studied. (3) The enzyme molecule responsible for RNA m^6^A modification in MYCN is still unknown. (4) Whether miR-98 is a new biomarker of NB remains to be explored.

The objective of the present investigation was to explore role of RNA N6-methyladenosine modification in miR-98/MYCN axis-mediated inhibition of neuroblastoma progression. In this study, we did not construct MYCN-MUT 3′UTR plasmid with the m6A site mutation in cells. It will be very interesting to construct MYCN mutant with the m6A site mutation for detecting the effect of m6A site mutation on MYCN mRNA or protein expression. However, we have done double-fluorescence reporter assay (Fig. 5B) by constructing MYCN-MUT sequences to address the scientific question of this study. The data in this study revealed that when miR-98 directly bound to MYCN 3′-UTR, it decreased MYCN expression (Fig. 3D, Fig. 4A). Therefore, it can be speculated that m6A site mutation affects the interaction between miR-98 and MYCN, which thus has an impact on MYCN mRNA or protein expression. Anyhow, it is RNA N6-methyladenosine modification, but not the mRNA or protein MYCN expression level alone is important in miR-98/MYCN axis-mediated inhibition of neuroblastoma progression.

In conclusion, the above results suggest that miR-98 can inhibit the malignant progress of neuroblastomas by targeting *MYCN* directly, and the interaction of these two molecules requires the participation of m^6^A modification.
